# Characteristics of revisits of children at risk for serious infections in pediatric emergency care

**DOI:** 10.1007/s00431-018-3095-0

**Published:** 2018-02-03

**Authors:** Evelien de Vos-Kerkhof, Dorien H. F. Geurts, Ewout W. Steyerberg, Monica Lakhanpaul, Henriette A. Moll, Rianne Oostenbrink

**Affiliations:** 1 Department of General Paediatrics, Erasmus MC-Sophia Children’s Hospital, Wytemaweg 80, 3015 Rotterdam, CN Netherlands; 2 Department of Public Health, Erasmus MC - University Medical Center Rotterdam, Rotterdam, Netherlands; 3 Department of Population, Policy and Practice UCL Great Ormond Street Institute of Child Health, Great Ormond Street, London, UK

**Keywords:** Emergency Department, Safety netting, Children, Revisit, Follow-up

## Abstract

In this study, we aimed to identify characteristics of (unscheduled) revisits and its optimal time frame after Emergency Department (ED) discharge. Children with fever, dyspnea, or vomiting/diarrhea (1 month–16 years) who attended the ED of Erasmus MC-Sophia, Rotterdam (2010–2013), the Netherlands, were prospectively included. Three days after ED discharge, we applied standardized telephonic questionnaires on disease course and revisits. Multivariable logistic regression analysis was used to identify independent characteristics of revisits. Young age, parental concern, and alarming signs and symptoms (chest wall retractions, ill appearance, clinical signs of dehydration, and tachypnea) were associated with revisits (*n* = 527) in children at risk for serious infections discharged from the ED (*n* = 1765). Children revisited the ED within a median of 2 days (IQR 1.0–3.0), but this was proven to be shorter in children with vomiting/diarrhea (1.0 day (IQR 1.0–2.0)) compared to children with fever or dyspnea (2.0 (IQR 1.0–3.0)).

*Conclusion*: Young age, parental concern, and alarming signs and symptoms (chest wall retractions, ill appearance, clinical signs of dehydration, and tachypnea) were associated with emergency health care revisits in children with fever, dyspnea, and vomiting/diarrhea. These characteristics could help to define targeted review of children during post-discharge period. We observed a disease specific and differential timing of control revisits after ED discharge.

**Table:** 

**What is Known** • *Fever, dyspnea, and vomiting/diarrhea are major causes of emergency care attendance in children.* • *As uncertainty remains on uneventful recovery, patients at risk need to be identified on order to improve safety netting after discharge from the ED.*
**What is New** • In children with fever, dyspnea, and vomiting/diarrhea, young age, parental concern and chest wall retractions, ill appearance, clinical signs of dehydration, and tachypnea help to define targeted review of children during the post-discharge period. • A revisit after ED discharge is disease-specific and seems to be shorter for children with vomiting/diarrhea than others.

**Electronic supplementary material:**

The online version of this article (10.1007/s00431-018-3095-0) contains supplementary material, which is available to authorized users.

## Introduction

Fever, dyspnea, and vomiting/diarrhea are major causes of emergency care attendance in childhood. Serious infections (SI) could be the underlying cause of these symptoms. Morbidity and mortality after Emergency Department (ED) visit due to serious infections or a complicated disease course of a self-limiting viral illness should not be underestimated. Infections account for 20% of childhood deaths in England, Wales, and Northern Ireland, with the greatest number in children aged 1–4 years [11, 14]. In the Netherlands, between 1969 and 2006, mortality due to infectious diseases compared to total childhood mortality was around 3.0% [22].

Serious infections are mostly defined as sepsis (including bacteremia), meningitis, pneumonia, osteomyelitis, cellulitis, gastroenteritis with severe dehydration, complicated urinary tract infection (positive urine culture and systemic effects such as fever), and viral respiratory tract infections complicated by hypoxia (e.g., bronchiolitis) [18]. At the ED, serious infections can be hard to recognize, as they can present similar to a self-limiting (viral) disease during early presentation, eventually leading to a diagnostic or treatment delay.

Dealing with this uncertainty on diagnosis or disease course after ED discharge, clinicians usually schedule revisits in a substantial number of cases. In addition, they provide parents with instructions on expected disease course, alarming signs and symptoms, and when to revisit, a well-known concept called “safety netting” [1, 16].

A recent systematic review concluded that studies concerning effects of safety netting interventions were mostly conflicting or with limited evidence [7]. They described strong associated characteristics of revisits as young children, infectious/respiratory symptoms, and progression of symptoms. However, evidence on follow-up management and its time frame as a part of the whole process of safety netting in children at risk for serious infections is lacking [10].

To improve the process of safety netting, we aimed to identify characteristics of (unscheduled) revisits and the timing of these revisits in a prospectively collected cohort of children with fever, vomiting/diarrhea, or dyspnea, discharged from the ED.

## Methods

### Study design and setting

We conducted a prospective follow-up study at the ED of the Erasmus MC-Sophia Children’s Hospital in Rotterdam. This large inner-city university hospital is visited annually by nearly 7000 children with a mixed ethnic population of which 35% have chronic comorbidity.

### Participants

We prospectively enrolled all consecutive children (≥ 1 month–< 16 years) attending the ED with fever, vomiting/diarrhea, or dyspnea from March 2010 to October 2013. Febrile children were defined as eligible if fever had been noted at home in the 24 h prior to presentation, when body temperature measured at the ED was ≥38.5 °C or fever was used as a positive discriminator of the Manchester Triage System (MTS) [6]. Children with vomiting/diarrhea needed to be suspected of a recent infectious cause. To be included, the illness episode had to be preceded by a minimum symptom free period of 2 weeks and the illness episode needed to be related to an infectious disease. Children were assigned to dyspnea when respiratory complaints, with or without bronchoconstriction, were the main reason of visiting the ED. Children with dyspnea who also suffered from fever were assigned to dyspnea if these symptoms took precedence over fever-related complaints. Given the aim of the study, i.e., improving discharge advice, we excluded children who were admitted to the hospital ward after initial ED visit. Next, as children with a known medical history or medical diagnosis get specific safety netting advice for their chronic condition in their outpatient follow-up (by pediatrician and specialist nurse), they are another population compared to the children with common acute illnesses at the ED. As children with a known medical history or medical diagnosis may be managed differently at ED or by (experienced) parents, we excluded children with complex needs as well as children with predefined asthma [24].

Revisit

### Data collection

All children who attended the ED were routinely triaged with the MTS. This digital recorded triage system is used to prioritize patients according to acuity [21]. In the analysis, MTS categories were reformatted into three categories: (1) emergent/very urgent, (2) urgent, and (3) non-urgent/standard to guarantee sufficient numbers per category. We collected patient characteristics from a structured electronic patient record system (gender, age, reason of ED visit, visit date, triage information), referral profile, duration of the complaints, clinical signs and symptoms, observations, and measures from physical examination (e.g., vital signs, temperature, breathing difficulty, clinical appearance) [13, 20, 24].

During the process of discharge, patients received information on alarming signs and symptoms, expected disease course, and on when and how to return. Part of the children received a scheduled revisit, by judgment of the attending physician, based on either the clinical signs and symptoms or expected complications, or on parental concern. In addition to these scheduled revisits (i.e., initiated/appointed by the physician at discharge), patients could revisit unscheduled (i.e., patient-initiated). These data were collected using a standardized telephonic questionnaire on the disease course which parents were asked to answer 3 days after ED discharge. The questionnaire included specifically data on duration or reoccurrence of symptoms as well as on complications, and on revisits to the hospital ED, to primary care, or to other health care settings. When the child was not yet fully recovered, we continued our follow-up by telephone until complete remission of their symptoms.

### Outcome measures and definitions

Our primary outcome measures were (1) revisits, defined as all revisits occurring for the same health care problem at either the GP (primary care) and the emergency department (secondary care) after the first ED visit, and (2) the time until this revisit, measured as the time gap between discharge and revisit. Secondary outcome measures included unscheduled revisits (defined as an unplanned control visits after the initial visit) and hospitalization following a revisit.

To evaluate parental concern, parents were asked if they considered their child’s illness at initial ED revisit to be different from earlier episodes. This is in accordance with previous studies in primary care, showing that parental concern is an important determinant of serious infections [17, 18].

### Ethics

Ethical approval was obtained from the institutional review board (IRB) of the Erasmus MC (MEC-2005-314). Informed consent was required and obtained from all parents.

### Statistical analysis

#### Variable selection

Variable selection for studying potential characteristics of revisits were based on previously published decision models or risk scores [2, 3, 8] and a recent systematic review on characteristics of pediatric health care revisits (Table 1) [10].

**Table 1:** Variable selection according to decision model, risk scores, and systematic review

	Feverkidstool [13]	Friedman dehydration score [9]	Indicators of dyspnea [15]	Systematic review [7]
Child characteristics				
Age	X			X
Gender	X			
Ill appearance	X	X		
Characteristics general				
Tachycardia	X			
Prolonged cap. Refill time	X			
Relevant medical history				X
Infectious/respiratory symptoms				X
Seizures				X
Progression/persistence of symptoms				X
CRP bedside (ln)	X			
Characteristics of fever				
Duration of fever	X			
Temperature (°C)	X			
Characteristics of dehydration				
Eyes		X		
Dry mucosa		X		
Tears		X		
Vomiting				
Characteristics of dyspnea				
Dyspnea			X	
Chestwall retractions	X		X	
Decreased oxygen saturation	X		X	
Tachypnea	X		X	
Auscultation			X	

#### Characteristics of revisits

Since patients with fever, vomiting/diarrhea, or dyspnea may differ in their disease course and time frame, we performed analysis separately for each of these patient groups. Time until ED revisit was evaluated by Kaplan-Meier survival analysis. Previous research in our setting showed that 12% of all discharged children underwent revisits of which 4% included interventions [4]. To analyze 10–15 characteristics of revisits, it was decided that we should have at least 10 times as many events (100–150 revisits) [4, 5]. With these distributions, we estimated to include at least around 830 (100/0.12) to 1250 (150/0.12) children.

#### Missing values

To allow optimal use of available data in multivariate models, missing data were imputed 10 times using a multiple imputation process with the mice algorithm in R software (version 3.0) under the assumption to be missing at random [26]. The imputation model included all variables which were considered in the multivariable logistic regression analysis, the outcome variable (revisits), and several relevant variables describing case mix of the patients (e.g., gender and MTS urgency) (Tables S1, and S2.1, S2.2, S2.3). All analyses, except for multiple imputation, were performed with SPSS software (version 20.0, SPSS Inc., Chicago).

## Results

**Fig. 1:**
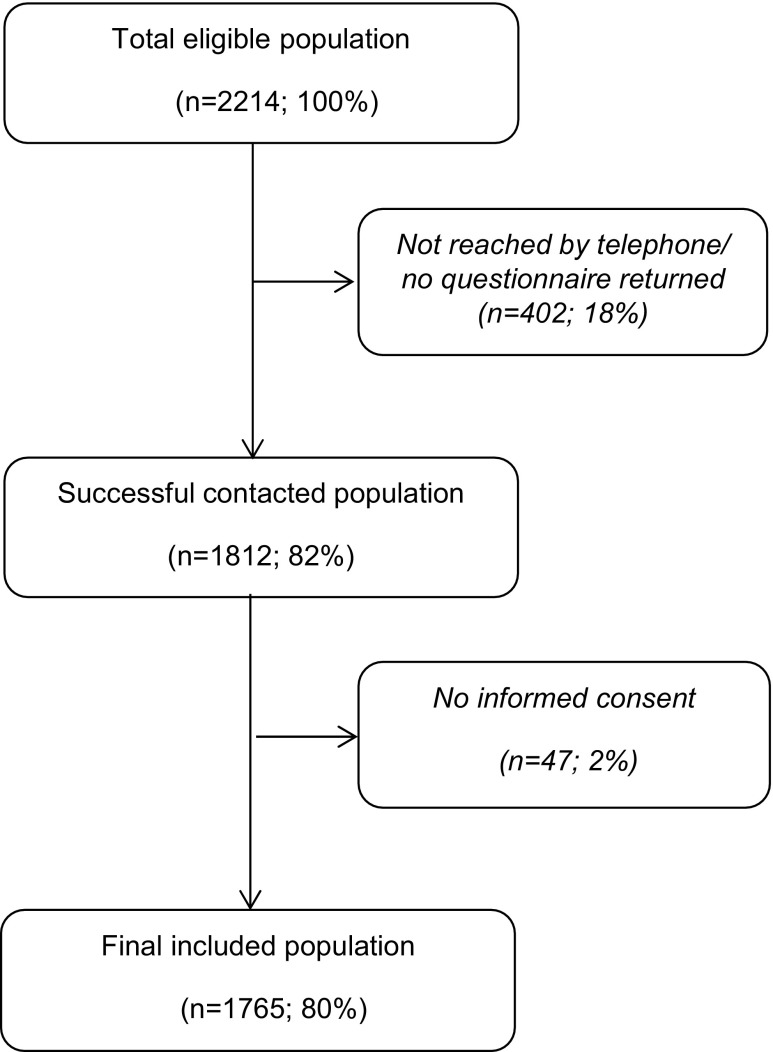
Patient flowchart

Successful follow-up by telephone was achieved for 1765 children, encompassing 80% of the total eligible population (*n* = 2214) (Fig. 1). Overall patients’ median age was 22 months (IQR 11–48), with 57% boys (*n* = 1000). The revisit rate was 30% (*n* = 527) and 3% (*n* = 54) of the children were hospitalized after revisiting the ED (Table 2). Most children were triaged as urgent patients (54%; *n* = 944) and 51% were referred by physicians.

**Table 2:** Demographics of total population

		Presenting problem, *n* (%)		
	Total, *n* = 1765 (100)	Fever, *n* = 1136 (64.4)	Vomiting/diarrhea, *n* = 372 (21.1)	Dyspnea, *n* = 257 (14.5)
Sex, male^a^	1000 (56.7)*	635 (55.9)	194 (52.2)	171 (66.5)
Age (months)^b^	22.0 (11.0–48.0)*	23.0 (12.0–51.0)	18.0 (9.0–41.0)	21.0 (8.0–41.0)
All revisits^a^	527 (29.9)	346 (30.5)	108 (29.0)	73 (28.4)
Unscheduled revisit^a^	352 (19.9)*	240 (21.1)	57 (15.3)	55 (21.4)
Secondary hospitalization	54 (3.1)	28 (2.5)	17 (4.6)	9 (3.5)
Parental concern	1410 (79.9)*	952 (83.8)	285 (76.6)	173 (67.3)
MTS urgency initial ED visit				
Emergent/very urgent	331 (18.7)*	203 (17.9)	34 (9.1)	94 (36.6)
Urgent	944 (53.5)*	700 (61.6)	161(43.3)	83 (32.3)
Standard/non-urgent	490 (27.8)*	233 (20.5)	177 (47.6)	80 (31.1)
Type of referred initial ED visit				
Self-referral	865 (49.0)*	547 (48.2)	216 (58.1)	102 (39.7)
Physician^c^	900 (51.0)*	589 (51.8)	156 (41.9)	155 (60.3)

*P value < 0.05

^a^Absolute number (percentage)

^b^Median (IQR)

^c^Including primary, secondary, and ambulance care

Febrile children constituted 64% (*n* = 1136) of included children, with 346 (*n* = 31%) revisits. Twenty-one percent (*n* = 372) children suffered from vomiting/diarrhea with 108 (29%) revisits. Fifteen percent (*n* = 257) of all children had dyspnea, with 73 (*n* = 28%) revisits.

### Characteristics of revisits overall

Out of 527 revisits, 352 (67%) revisits were unscheduled (Table 3). The number of unscheduled revisits was the lowest for children with vomiting/diarrhea (*n* = 57, 15.3%) and the highest for children with dyspnea (*n* = 55, 21.4%) (Table 3).

**Table 3:** Characteristics of revisits

	Presenting problem, *n* (%)			
	Total, *n* = 527/1765 (29.9)	Fever, *n* = 346/1136 (30.5)	Vomiting/diarrhea, *n* = 108/372 (29.0)	Dyspnea, *n* = 73/257 (28.4)
Revisits with intervention^a^	293 (55.6)^d^	199 (57.5)	47 (43.5)	47 (64.4)
Unscheduled^a^	352 (66.8)^d^	240 (69.4)	57 (52.8)	55 (75.3)
Time until revisit (days)^b^	2.0 (1.0–3.0)	2.0 (1.0–3.0)	1.0 (1.0–2.0)	2.0 (1.0–4.0)^c^
Time until unscheduled revisit	3.0 (2.8–3.2)	2.0 (1.7–2.3)	2.0 (1.4–2.6)	3.0 (2.4–3.6)
Setting of revisit				
Primary care	249 (47.2)	180 (52.0)	35 (32.4)	34 (46.6)
Emergency care	278 (52.8)	166 (48.0)	73 (67.6)	39 (53.4)

^a^Absolute number (percentage)

^b^Median (IQR)

^c^Log rank < 0.000

^d^*P* value < 0.05:

- More revisits with intervention in febrile children versus children with vomiting/diarrhea (chi-square)

- More unscheduled revisits in febrile children versus children with vomiting/diarrhea (chi-square)

- More prescribed antibiotics in febrile children versus children with vomiting/diarrhea and children with dyspnea (chi-square)

- More prescribed airway medicine in children with dyspnea versus febrile children and children with vomiting/diarrhea (chi-square)

- More prescribed gastro-intestinal medicine in children with vomiting/diarrhea versus febrile children and children with dyspnea (chi-square)

Children revisited the ED after a median of 2 days (IQR 1.0–3.0). Children with vomiting/diarrhea revisited the ED significantly at a shorter interval (1.0 day (IQR 1.0–2.0)) than children with fever or dyspnea (2.0 (IQR 1.0–3.0)) (log rank *p* < 0.0001).

### Characteristics of revisits of febrile children

Age, parental concern, and chest wall retractions were associated with revisits in febrile children (multivariable ORs between 1.30–1.98) (*p* value < 0.1) (Table 4). Young age and parental concern, in particular, were associated with unscheduled revisits (respectively, OR (CI 95%) 1.42 (1.04–1.95) and OR (CI 95%) 1.81 (1.13–2.90)) **(**Table 4**)**.

**Table 4:** Determinants of revisits in children with fever, vomiting/diarrhea, and dyspnea

Determinants	Revisits, *n* = 346	Unscheduled revisits, *n* = 240
Fever	OR (95% CI)	OR (95% CI)
Age < 1 year	1.30 (0.98–1.72)*	1.42 (1.04–1.95)**
Parental concern	1.71 (1.15–2.55)**	1.81 (1.13–2.90)**
Chestwall retractions	1.98 (1.02–3.82)**	1.68 (0.82–3.44)
Vomiting/diarrhea	*n* = 108	*n* = 57
Age < 1 year	1.87 (1.14–3.07)**	2.09 (1.15–3.80)**
Ill appearance	1.91 (1.03–3.53)**	1.29 (0.58–2.85)
Clinical signs of dehydration	2.26 (1.12–4.53)**	1.96 (0.85–4.54)
Tachypnea	5.08 (2.30–11.25)**	4.12 (1.59–10.69)**
Any sign of dyspnea	*n* = 73	*n* = 55
Age < 3 year	0.58 (0.31–1.09)*	0.58 (0.28–1.17)

*Significant predictors (*p* < 0.10), **significant predictors (*p* < 0.05)

### Characteristics of revisits of children with vomiting/diarrhea

The characteristics age < 1 year, ill appearance, clinical signs of dehydration at initial assessment, and tachypnea were associated with revisits (*p* value < 0.10) **(**Table 4**)**. Age and tachypnea remained strongly independent associated with unscheduled revisits **(**Table 4**)**.

### Characteristics of revisits of children with dyspnea

In children with dyspnea, we could only identify the determinant “age < 3 years” to be significantly associated with revisits (*p* value < 0.10) **(**Table 4**)**.

## Discussion

### Main findings

In a prospectively study on clinical symptoms and signs that are associated with health care revisits in children with fever, dyspnea, and vomiting/diarrhea, we observed young age, parental concern, and alarming signs and symptoms (chest wall retractions, ill appearance, clinical signs of dehydration, and tachypnea) to be the most important. Children with vomiting/diarrhea revisited the ED at a shorter interval (median 1 day; IQR 1.0–2.0) compared with children with fever or dyspnea (median 2 days; IQR 1.0–4.0).

### Clinical implications and comparison with other studies

In order to optimize the process of safety netting, we prospectively evaluated characteristics of revisits in children at risk for serious infections discharged from the ED, originating from the question on which children need revisits and in what time frame. Although we identified various characteristics, they do not select a definite population that will not (need to) revisit the ED.

In summary, there is a need for safety netting in all children after discharge from the ED, however, with special attention for a subgroup of children at risk with young age, parental concern, and specific symptoms and signs. Our results support specific time frames for specific presenting conditions.

### Strengths and limitations

The major strength of this prospective study is the large number of children with complete follow-up, as we included up to 80% of the eligible children successfully in our study. Second, our study did not only include revisits to our hospital ED, but also to other EDs in the area as well as revisits to primary care or other emergency care settings.

Lastly, we studied the role of parental concern in the emergency care setting [17, 18]. We found an association between parental concern and revisits only in the group of febrile children. In the majority of affirmative answers, parental concern was caused by a longer duration or a more severe illness. It is important to remark this indicator of a probable complicated clinical course, as it emphasizes the meaningful role of parents in the assessment of their child’s illness in secondary care settings in addition to the known role in primary care [18].

This study has some limitations. In our study, we chose revisits as our primary outcome measure and we separately analyzed unscheduled revisits. As former research showed the following risk factors for pediatric ED revisits: arrival in the evening, respiratory diagnosis, and acute triage category [19], one might argue that our secondary outcome, i.e., unscheduled revisit and hospitalization, would be of more clinical relevance. However, unscheduled revisits can be influenced by the clinical setting and by the time frame the scheduled revisit was originally planned in, and also would be related to parental background and concepts of disease and their uncertainty or comprehension ability to understand provided information.

There are several factors, influencing the attending physician’s decision to schedule a follow-up appointment or to admit a patient, besides having to perform further diagnostic tests or treatment. We observed 293/527 (55.6%) visits with an intervention (defined as diagnostics, treatment, or admission) (Table 3). Admission occurred in 54 (10.2%) patients.

In all other revisits (234/527; 44.4%) patients did not receive any diagnostics or treatment, nor were they admitted to the hospital. However, to regard them just as a “reassurance”-revisit for parents would be too simplistic, as other factors like alarming signs, gut feeling, and experience of the attending physician can influence this decision. We can only speculate about the reasons as detailed information is missing, and this topic was beyond the scope of our study.

Selection bias and recall bias are well-known problems of questionnaire studies [12, 23]. However, our study reached a high response rate of 80%, in contrast to most response rates of telephonic or postal questionnaire studies of less than 60% [25]. Recall bias may especially have influenced the subjective determinant parental concern. However, as parents were called only 3 days after ED discharge, this should be less of a problem in our study.

## Conclusion

In this prospective cohort study on ED patients, we observed young age, parental concern, and alarming signs and symptoms (chest wall retractions, ill appearance, clinical signs of dehydration, and tachypnea) being associated with emergency health care revisits in children with fever, dyspnea, and vomiting/diarrhea. In addition to the general need for safety netting procedures in children at risk for serious infections, these characteristics could help to define targeted review of children during post-discharge period. A control visit after ED discharge is disease-specific, and the post-discharge interval seems to be shorter for children with vomiting/diarrhea than others in particular.

## Electronic supplementary material

ESM 1 (DOCX 17 kb)

ESM 2 (DOCX 16 kb)

ESM 3 (DOCX 15 kb)

ESM 4 (DOCX 15 kb)

## Abbreviations

AUC: Area under the receiver operating characteristic curve
ED: Emergency Department
IQR: Interquartile range
MTS: Manchester Triage System
SI: Serious infections

## References

[1] Almond S, Mant D, Thompson M (2009). Diagnostic safety-netting. Br J Gen Pract.

[2] Broom M (2007). Physiology of fever. Paediatr Nurs.

[3] Bruyninckx R, Van den Bruel A, Aertgeerts B, Van Casteren V, Buntinx F (2008). Half of the patients with chest pain that are urgently referred are transported in unsafe conditions. Eur J Emerg Med.

[4] Bruyninckx R, Van den Bruel A, Aertgeerts B, Van Casteren V, Buntinx F (2009). Why does the general practitioner refer patients with chest pain not-urgently to the specialist or urgently to the emergency department? Influence of the certainty of the initial diagnosis. Acta Cardiol.

[5] Bruyninckx R, Van den Bruel A, Hannes K, Buntinx F, Aertgeerts B (2009). GPs’ reasons for referral of patients with chest pain: a qualitative study. BMC Fam Pract.

[6] de Vos-Kerkhof E, Nijman RG, Vergouwe Y, Polinder S, Steyerberg EW, van der Lei J, Moll HA, Oostenbrink R (2015). Impact of a clinical decision model for febrile children at risk for serious bacterial infections at the emergency department: a randomized controlled trial. PLoS One.

[7] de Vos-Kerkhof E, Geurts DH, Wiggers M, Moll HA, Oostenbrink R (2016). Tools for “safety netting” in common paediatric illnesses: a systematic review in emergency care. Arch Dis Child.

[8] Digenio AG, Sim JG, Krige K, Stewart A, Morris R, Dowdeswell RJ, Padayachee GN (1991). The Johannesburg cardiac rehabilitation programme. S Afr Med J.

[9] Goldman RD, Friedman JN, Parkin JC (2008) Validation of the Clinical dehyadrtaion Scale for children with acute gastroenteritis. Pediatrics 122:545–549 10.1542/peds.2007-3141 18762524

[10] Habenicht BF, Craig CF, Prezhdo OV (2006). Time-domain ab initio simulation of electron and hole relaxation dynamics in a single-wall semiconducting carbon nanotube. Phys Rev Lett.

[11] Jaeschke R, Guyatt GH, Sackett DL (1994). Users’ guides to the medical literature. III. How to use an article about a diagnostic test. B. What are the results and will they help me in caring for my patients? The Evidence-Based Medicine Working Group. JAMA.

[12] Marques WS, Menor Ede A, Sial AN, Manso VA, Freire SS (2007). Oceanographic parameters in continental margin of the State of Ceara (northeastern Brazil) deduced from C and O isotopes in foraminifers. An Acad Bras Cienc.

[13] Nijman RG, Vergouwe Y, Thompson M, van Veen M, van Meurs AH, van der Lei J, Steyerberg EW, Moll HA, Oostenbrink R (2013). Clinical prediction model to aid emergency doctors managing febrile children at risk of serious bacterial infections: diagnostic study. BMJ.

[14] Oostenbrink R, Moons KG, Donders AR, Grobbee DE, Moll HA (2001). Prediction of bacterial meningitis in children with meningeal signs: reduction of lumbar punctures. Acta Paediatr.

[15] Qureshi F Pestian J davis P Zaritsky A (1998) Effect of nebulized ipratropium in the hospitalizateion rates of children with astma. N Eng J Med 339:1030–1035 10.1056/NEJM199810083391503 9761804

[16] Roland D, Jones C, Neill S, Thompson M, Lakhanpaul M (2014). Safety netting in healthcare settings: what it means, and for whom?. Arch Dis Child Educ Pract Ed.

[17] Van den Bruel A, Aertgeerts B, Bruyninckx R, Aerts M, Buntinx F (2007). Signs and symptoms for diagnosis of serious infections in children: a prospective study in primary care. Br J Gen Pract.

[18] Van den Bruel A, Haj-Hassan T, Thompson M, Buntinx F, Mant D, European Research Network on Recognising Serious Infection i (2010). Diagnostic value of clinical features at presentation to identify serious infection in children in developed countries: a systematic review. Lancet.

[19] van der Linden MC, Lindeboom R, de Haan R, van der Linden N, de Deckere ER, Lucas C, Rhemrev SJ, Goslings JC (2014). Unscheduled return visits to a Dutch inner-city emergency department. Int J Emerg Med.

[20] van Ierland Y, Seiger N, van Veen M, Moll HA, Oostenbrink R (2013). Alarming signs in the Manchester triage system: a tool to identify febrile children at risk of hospitalization. J Pediatr.

[21] van Veen M, Steyerberg EW, Ruige M, van Meurs AH, Roukema J, van der Lei J, Moll HA (2008). Manchester triage system in paediatric emergency care: prospective observational study. BMJ.

[22] Veldhoen ES, Wolfs TF, van Vught AJ (2009). Changes in infectious disease mortality among children in the Netherlands. Eur J Pediatr.

[23] Yuan W, Craig S, Si Z, Farzan M, Sodroski J (2004). CD4-induced T-20 binding to human immunodeficiency virus type 1 gp120 blocks interaction with the CXCR4 coreceptor. J Virol.

[24] Zachariasse JM, Kuiper JW, de Hoog M, Moll HA, van Veen M (2016). Safety of the Manchester triage system to detect critically ill children at the emergency department. J Pediatr.

[25] Zhang P, Wu J, Wang Z, Sibata C (2002). Considerations for the implementation of target volume protocols in radiation therapy: in regard to Craig et al., IJROBP 2001;49:241-250. Int J Radiat Oncol Biol Phys.

[26] Zoraghi R, See RH, Axerio-Cilies P, Kumar NS, Gong H, Moreau A, Hsing M, Kaur S, Swayze RD, Worrall L, Amandoron E, Lian T, Jackson L, Jiang J, Thorson L, Labriere C, Foster L, Brunham RC, McMaster WR, Finlay BB, Strynadka NC, Cherkasov A, Young RN, Reiner NE (2011). Identification of pyruvate kinase in methicillin-resistant *Staphylococcus Aureus* as a novel antimicrobial drug target. Antimicrob Agents Chemother.

