# Supplementary material for: Characteristics of revisits of children at risk for serious infections in pediatric emergency care
Source: Eur J Pediatr. 2018 Feb 3;177(4):617–24. doi: 10.1007/s00431-018-3095-0 (PMC5851682; doi:10.1007/s00431-018-3095-0)
Supplement: Supplementary file 1 — (DOCX 17 kb) [file 431_2018_3095_MOESM1_ESM.docx]

**Supplementary file 1: missing data per patient category**

|  |  |  |  |  |
| --- | --- | --- | --- | --- |
|  |  |  |  |  |
|  |  | FEVER | VOMITING/DIARRHOEA | DYSPNOEA |
| DETERMINANTS |  | n=1136 | n=372 | n=257 |
|  |  | (100%) | (100%) | (100%) |
|  |  |  |  |  |
|  |  |  |  |  |
| *Patient characteristics* |  |  |  |  |
| Age |  | 0 | 0 | 0 |
| Gender (male) |  | 0 | 0 | 0 |
|  |  |  |  |  |
| *Disease characteristics* |  |  |  |  |
| Parental concern |  | 18 (1.6) | 34 (9.1) | 8 (3.1) |
| Duration of fever/ illness |  | 175 (15.4) | 40 (10.8) | 22 (8.6) |
| Ill appaerance |  | 48 (4.2) | 28 (7.5) | 25 (9.7) |
| Temperature (°C) |  | 7 (0.6) | 6 (1.6) | 19 (7.4) |
| Tachypnoea |  | 140 (12.3) | 79 (21.2) | 19 (7.4) |
| Tachycardia |  | 106 (9.3) | 34 (9.1) | 18 (7.0) |
| Decreased oxygen saturation |  | 286 (25.2) | 237 (63.7) | 8 (3.1) |
| Prolonged cap. refill time (peripheral) |  | 41 (3.6) | 48 (12.9) | 37 (14.4) |
| Chestwall retractions |  | 217 (19.1) |  | 20 (7.8) |
| *Diagnostics* |  |  |  |  |
| CRP bedside (ln) |  | 419 (36.9) | 277 (74.5) | 210 (81.7) |
| Less eating |  | - | 49 (13.2) | - |
| Decreased urine output |  | - | 90 (24.2) | - |
| Dry mucous membranes |  | - | 40 (10.8) | - |
| Sunken fontanel |  | - | 86 (23.1) | - |
| Decreased turgor |  | - | 66 (17.7) | - |
| Decreased tears |  | - | 115 (30.9) | - |
| Thirsty |  | - | 200 (53.8) | - |
| Sunken eyes |  | - | 78 (21.0) | - |
| Vomiting |  | - | 52 (14.0) | - |
| Coughing |  | - | - | 33 (12.8) |
| Stridor |  | - | - | 20 (7.8) |
| Nasal flairing |  | - | - | 49 (19.1) |
| Groaning |  | - | - | 62 (24.1) |
| Auscultation |  | - | - | 16 (6.2) |

0 no missing data

- no relevant determinant regarding the presenting problem
