# Supplementary material for: Characteristics of revisits of children at risk for serious infections in pediatric emergency care
Source: Eur J Pediatr. 2018 Feb 3;177(4):617–24. doi: 10.1007/s00431-018-3095-0 (PMC5851682; doi:10.1007/s00431-018-3095-0)
Supplement: Supplementary file 2 — (DOCX 16 kb) [file 431_2018_3095_MOESM2_ESM.docx]

**Supplementary file 2: variable selection**

*Table 2.1: multivariable regression analysis in children with fever*

| DETERMINANTS | REVISITS  *n=346* |  |
| --- | --- | --- |
|  |  |  |
| *Patient characteristics*  *n=1136* | *OR*  *(95% CI)* | *n/total (%)* |
| *Age* |  |  |
| 0-3m | 1.23 (0.56-2.69) | 12 (3.5) |
| 3-6m | 1.02 (0.57-1.84) | 23 (6.6) |
| 6-12m | 1.35 (0.88-2.07)*^*^* | 74 (21.3) |
| 1-5y | 0.96 (0.67-1.36) | 175 (50.6) |
| >5y (REF) | *ref* | 62 (17.9) |
| Age <1y | 1.31 (0.96-1.77)*^*^* | 109 (31.5) |
| Age <3y |  |  |
| Age <5y | 1.05 (0.74-1.49) | 284 (82.1) |
| Gender (male) | 1.24 (0.95-1.62)*^*^* | 208 (60.1) |
|  |  |  |
| *Disease characteristics* |  |  |
| Parental concern | 1.73 (1.15-2.60)*^*^* | 310 (89.6) |
| Duration of fever | 1.00 (0.92-1.09) | *continuous* |
| Ill appearance | 1.05 (0.74-1.48) | 79 (22.8) |
| Temperature (°C) | 1.07 (0.93-1.24) | *continuous* |
| Tachypnoea | 1.19 (0.86-1.64) | 105 (30.3) |
| Tachycardia | 1.17 (0.85-1.61) | 114 (32.9) |
| Decreased oxygen saturation | 0.57 (0.08-4.28) | 6 (1.7) |
| Prolonged cap. refill time (peripheral) | 1.30 (0.77-2.19) | 28 (8.1) |
| Chestwall retractions | 1.82 (0.94-3.54)*^*^* | 32 (9.2) |
|  |  |  |
| *Diagnostics* |  |  |
| CRP bedside (ln) | 1.00 (1.00-1.00) | *continuous* |
|  |  |  |

*^*^significant determinants (p<0.20)*
