# Supplementary material for: Characteristics of revisits of children at risk for serious infections in pediatric emergency care
Source: Eur J Pediatr. 2018 Feb 3;177(4):617–24. doi: 10.1007/s00431-018-3095-0 (PMC5851682; doi:10.1007/s00431-018-3095-0)
Supplement: Supplementary file 3 — (DOCX 15 kb) [file 431_2018_3095_MOESM3_ESM.docx]

*Table 2.2: multivariable regression analysis in children with vomiting and diarrhoea*

| DETERMINANTS | REVISITS  *n=108* |  |
| --- | --- | --- |
|  |  |  |
| *Patient characteristics*  *n=372* | *OR*  *(95% CI)* | *n/total (%)* |
| Age |  |  |
| 0-3m | 2.54 (0.72-9.00)*^*^* | 6 (5.6) |
| 3-6m | 2.10 (0.70-6.31)*^*^* | 13 (12.0) |
| 6-12m | 3.03 (1.15-7.99)*^*^* | 33 (30.6) |
| 1-5y | 1.61 (0.68-3.84) | 44 (40.7) |
| >5y (REF) | *Ref* | 12 (11.1) |
| Age <1y | 1.59 (0.89-2.83)*^*^* | 52 (48.1) |
| Age <3y | 1.29 (0.47-3.51) | 88 (81.5) |
| Age <5y | 1.32 (0.43-4.12) | 96 (88.9) |
| Gender (male) | 1.08 (0.63-1.87) | 60 (55.5) |
|  |  |  |
| *Disease characteristics* |  |  |
| Parental concern | 1.46 (0.65-3.30) | 97 (89.8) |
| Duration of fever | 1.06 (0.92-1.23) | *continuous* |
| Ill appaerance | 1.89 (0.96-3.72)*^*^* | 32 (29.6) |
| Temperature (°C) | 1.28 (0.91-1.78)*^*^* | *continuous* |
| Tachypnoea | 4.85 (2.19-10.72)*^*^* | 35 (32.4) |
| Tachycardia | 0.52 (0.20-1.38)*^*^* | 12 (11.1) |
| Prolonged cap. refill time (peripheral) | 0.83 (0.36-1.94) | 17 (15.7) |
| Signs of dehydration | 2.21 (1.08-4.52)*^*^* |  |
| *Diagnostics* |  |  |
| CRP bedside (ln) | 1.01 (0.99-1.02) | *continuous* |
|  |  |  |

*^*^significant determinants (p<0.20)*
