# Supplementary material for: Characteristics of revisits of children at risk for serious infections in pediatric emergency care
Source: Eur J Pediatr. 2018 Feb 3;177(4):617–24. doi: 10.1007/s00431-018-3095-0 (PMC5851682; doi:10.1007/s00431-018-3095-0)
Supplement: Supplementary file 4 — (DOCX 15 kb) [file 431_2018_3095_MOESM4_ESM.docx]

*Table 2.3: multivariable regression analysis in children with dyspnea*

| DETERMINANTS | REVISITS  *n=73* |  |
| --- | --- | --- |
|  |  |  |
| *Patient characteristics*  *n=257* | *OR*  *(95% CI)* | *n/total (%)* |
| Age |  |  |
| 0-3m | 2.59 ( 0.64-10.53)*^*^* | 7 (9.6) |
| 3-6m | 1.40 (0.40-4.88) | 9 (12.3) |
| 6-12m | 1.85 (0.56-6.07) | 13 (17.8) |
| 1-5y | 1.64 (0.61-4.40) | 36 (49.3) |
| >5y (ref) | *ref* | 8 (11.0) |
| Gender (male) | 1.55 (0.80-3.00)*^*^* | 54 (74.0) |
|  |  |  |
| *Disease characteristics* |  |  |
| Parental concern | 1.08 (0.54-2.17) | 52 (71.2) |
| Ill appaerance | 0.79 (0.28-2.19) | 10 (13.7) |
| Tachypnoea | 1.52 (0.78-2.96) | 42 (57.5) |
| Tachycardia | 1.19 (0.55-2.55) | 18 (24.7) |
| Decreased oxygen saturation | 0.81 (0.11-5.79) | 3 (4.1) |
| Chestwall retractions | 0.81 (0.37-1.76) | 34 (46.6) |
| Coughing | 1.06 (0.44-2.52) | 60 (82.2) |
| Stridor | 1.18 (0.48-2.90) | 20 (27.4) |
| Nasal flairing | 0.85 (0.26-2.82) | 10 (13.7) |
| Groaning | 0.80 (0.22-2.88) | 10 (13.7) |
| Auscultation | 0.67 (0.34-1.32) | 38 (52.1) |
|  |  |  |
| *Diagnostics* |  |  |
| CRP bedside (ln) | 1.16 (0.66-2.03) | *continuous* |
|  |  |  |

*^*^significant determinants (p<0.20)*
